# Was COVID-19 an unexpected catalyst for more equitable learning outcomes? A comparative analysis after two years of disrupted schooling in Australian primary schools

**DOI:** 10.1007/s13384-023-00614-y

**Published:** 2023-03-01

**Authors:** Andrew Miller, Leanne Fray, Jennifer Gore

**Affiliations:** Teachers and Teaching Research Centre, School of Education, University of Newcastle, Callaghan, Australia

**Keywords:** Student achievement, COVID-19, Pandemic, Public school, Primary education

## Abstract

By the end of 2021, more than 168 million students across the globe had missed a year of face-to-face schooling due to the COVID-19 pandemic. In NSW, Australia, most students engaged in learning from home for eight weeks during 2020 and a further 14 weeks during 2021. This study provides robust empirical evidence on how two years of disruptions to schooling affected student learning. Drawing on matched data for 3,827 Year 3 and 4 students from 101 NSW government schools, this paper compares student achievement growth in mathematics and reading for 2019 (pre-pandemic) and 2021 (second year of the pandemic) student cohorts. While overall there was no significant difference between cohorts, when analysed by socio-educational advantage, we were surprised to find that students in the lowest band achieved approximately three months’ additional growth in mathematics. Arguably, grave concerns about the potentially dire impact of COVID-19 on the learning of disadvantaged students were met by investments that made a difference. We argue that targeted funding and system-wide initiatives to support more equitable outcomes should remain a priority after the pandemic if Australia is to meet its aspirations for excellence and equity.

## Introduction

The unprecedented disruption to schooling caused by the COVID-19 pandemic impacted more than 90% of school students, affecting the lives of more than 1.5 billion children globally (Psacharopoulos et al., 2020; United Nations Educational, Scientific and Cultural Organization [UNESCO], 2020). In almost all countries, schools closed and reopened multiple times in order to contain the spread of the virus (UNESCO & International Association for the Evaluation of Educational Achievement, 2022). By the beginning of 2022, more than 616 million students worldwide remained affected by school closures (UNICEF, 2022). This widespread interruption to schooling raised significant alarm, globally, about the short- and longer-term impact on student learning (Burgess & Sievertsen, 2020; Hampshire, 2020) and student wellbeing. Our focus in this paper is the effects of COVID-19 on student achievement after two years of disrupted schooling.

Gauging the effects of COVID-19 has been limited by the available evidence. Early modelling of predicted effects, based on previous studies of disruptions to schooling, cautioned that students would be behind in their learning by as much as 1.1 years (see for example, Azevedo et al., 2021; Haeck & Larose, 2022; Hevia et al., 2022; Kuhfeld et al., 2020a, 2020b). However, most prior studies have focused on short-term, localised school closures due to natural disasters and school shootings, rather than the system-wide school closures that have characterised the pandemic.

Empirical studies of the *actual* impact of COVID-19 on student learning did not begin to appear in academic publications until 2021. However, as reported in two early systematic reviews, very few studies have used robust comparative data collected prior to COVID-19 as a basis for claims about the impact of school closures (Donnelly & Patrinos, 2021; Hammerstein et al., 2021). Of the available studies that do include comparable measures, most provide evidence of negative effects on student achievement. Learning loss was reported, for instance, in nine of the 11 studies included in Hammerstein’s (2021) systematic review (effect size − 0.37 *SD* to − 0.03 *SD*) (Hammerstein et al., 2021), and Patrinos et al. (2022) reported learning loss of 0.17 standard deviations on average from 36 robust studies.

Notably, these negative effects have been disproportionately borne by students in disadvantaged settings (Catalano et al., 2021). For example, a Belgian study of more than 4000 primary school students found greater learning losses in mathematics in schools with a higher proportion of disadvantaged students (Maldonado et al., 2020). Similarly, a study of 350,000 primary school students in the Netherlands reported greater decline in achievement for students from homes with lower levels of education (Engzell et al., 2021). Likewise, a study from the United States, involving approximately 7 million students across Grades 3–8, found students in high poverty schools were disproportionately impacted (Lewis et al., 2021).

In yet other studies, however, the impact of COVID-19 disruptions to schooling has been less clear-cut. In our Australian comparison of more than 4800 Year 3 and 4 students between 2019 and 2020, we found Year 3 students from the least advantaged schools displayed 2 months’ less growth in mathematics, but no difference in reading (Gore et al., 2021a). In Germany, lower achieving students were also found to be more affected in mathematics while higher achieving students were more affected in reading (Schult et al., 2022). A study of more than 28,000 students in Switzerland found that students were largely unaffected by school closures, although the learning of primary school students was found to have slowed (Tomasik et al., 2021). In the Netherlands, the use of adaptive practising software during school closures led to faster progress in mathematics for primary school students in a study involving 53,0000 students (Meeter, 2021).

Published analyses using pre- and post- COVID-related school closure data remain relatively scarce but important in identifying the impacts of COVID-19 disruption on student outcomes (academic or otherwise). To date, most studies report on the effects during the first affected school year and we are not aware of any studies (at the time of writing) that examine the impact on student academic achievement of the second year of pandemic school closures in Australia. This paper is unique, then, in reporting results of such a study, thus contributing new insights to global understanding of how student achievement has been affected *over time*.

### Australian school students and COVID-19 school closures

Australia is renowned for being relatively unscathed by COVID-19, but students in Australia (especially in the states of Victoria and NSW) were subject to multiple learning from home episodes during government lockdowns. In Victoria, students engaged in learning from home for more than 20 weeks in 2020 and a further 14 weeks in 2021. In New South Wales (NSW), the location of our study, students learned from home for up to eight weeks in the 2020 school year and up to an additional 14 weeks in 2021. During both periods, teachers delivered lessons via a combination of online learning, paper-based learning packs, and face-to-face schooling for the children of essential workers (Fray et al., 2022a).

While Australian studies have examined the impact of COVID-19 school closures on students living in disadvantaged circumstances (Broerse, 2021), on student wellbeing (Fray et al., 2022a; Lyons et al., 2021) and on physical activity and screen time (Nathan et al., 2021; Reece et al., 2021), limited empirical evidence is available on student academic achievement. There are a few exceptions. An Education Department study involving more than 62,000 Year 3 NSW school students found test results aligned with the expected trajectory for numeracy, but were three to four months behind in reading (NSW Department of Education, 2020). In contrast, we found no significant differences, overall, between 2019 and 2020 in student achievement in mathematics or reading (Gore et al., 2021a). However, as noted above, when the data were examined by year level (grade) and school-level advantage, Year 3 students in less advantaged schools displayed less growth in mathematics, but not reading. Further, no significant differences were identified for Year 4 students or other equity categories (including First Nations students and students in regional and remote locations). The substantial differences between these two studies could be attributed to major differences in the student samples, testing instruments, and protocols used. We are aware that other states carried out their own testing but, to date, the results do not appear to have been made public.

The only other source of systematic data on changes to student achievement in Australia comes from the annual national assessment programme (NAPLAN), the dominant mechanism for tracking and benchmarking student learning in mathematics and literacy. However, NAPLAN testing was cancelled in 2020 to reduce pressure on students and teachers already grappling with the shock of the pandemic (Australian Curriculum, Assessment and Reporting Authority [ACARA], 2020a). The testing in Term 2 each year means that NAPLAN would, in any event, have been too early to illuminate the impact of the pandemic. Results of the 2021 NAPLAN testing revealed no significant decline in literacy and numeracy at any learning stage (ACARA, 2021). This result aligns with our own analysis at the whole sample level. A more fine-grained analysis of the data to examine the possible differential impact on students from disadvantaged backgrounds is not available (Sonnemann & Hunter, 2021).

In short, the limited evidence on what happened to student learning in Australia during the first year of the pandemic paints a reasonably promising picture. Certainly, dire predictions of ‘learning loss’ (Sonnemann & Goss, 2020) were not borne out in the evidence.

## Government boosts to schooling

Given widespread concern, globally, about potentially catastrophic effects of COVID-19, state governments quickly implemented policies designed to boost student learning. In NSW, an additional $337 million was provided in 2021 to support small group tutoring across schools in NSW (NSW Government, 2020c). This support was extended in 2022, with another $383 million to support students whose learning had been most affected (based on time spent away from school and level of disadvantage in the school) (NSW Government, 2021d). In the smaller state of Victoria, $250 million was provided for a tutor learning initiative in early 2021. An additional $230 million was allocated for the 2022 school year to provide targeted learning support to students who struggled the most with remote learning (Victorian Government, 2022). While Queensland has pledged $100 million over three years to support student wellbeing, other Australian states with more limited school closures have not (at the time of writing) taken this path.

In NSW, this funding was accompanied by a raft of other initiatives designed to support student learning. These included new supports for teachers such as a time-saving hub with resources for lesson planning (NSW Government, 2021a) and ‘Check in’ assessments to assist teachers in assessing, planning, and tailoring specific support for students (NSW Government, 2020a). A new web series was developed, EducationLive, to engage students in learning as educators, artists, athletes, and industry experts (for example, popular children’s entertainer, Emma Wiggle) delivered lessons to children via live video each morning (NSW Government, 2021c). Guided learning packs were made available to teachers, parent/carers, and students during learning from home (NSW Government, 2021b). Network infrastructure was upgraded to provide faster and more reliable Internet access to schools (NSW Government, 2020b). And, importantly, a student wellbeing programme added 100 school-based nurses to schools across the state (NSW Government, 2020d). A host of changes to schooling were also enacted, such as mandatory mask wearing and restrictions on school excursions, inter-school sport, and teacher professional development activities.

Given all these government interventions designed to support teachers and students during the pandemic, we were interested in investigating their impact on student learning. While we all hoped the pandemic would be over, and life and schooling would return to normal (or better) in 2021, this was not the case. There was deep concern in the community about the cumulative effects of COVID-19 on student learning (Tudge, 2021; UNICEF, 2021), especially for younger students whose critical formative years had been affected. In Australia and internationally, no study to date has reported how student learning was affected by two consecutive years of disruptions to schooling. This is partly because, in Australia, the school year commences in late January and concludes in mid-December whereas, in many other countries, the second year of schooling affected by the pandemic had not finished.

In the study reported here, we compared the academic achievement of a cohort of students from 2021 with a matched cohort from 2019 to examine the potential effects of two years of disrupted schooling for students in Years 3 and 4 in NSW primary schools. As with our previous analysis of the effects of COVID-19 in 2020 (Gore et al., 2021a), we did not set out to study the effects of the pandemic on student achievement. In 2021, we were in the middle of collecting data for a randomised controlled trial (RCT) when the Delta variant emerged in Australia and, once again, forced students to learn from home. By this time, we had pre-intervention data collected in February and March 2021. We knew our intervention would be affected by the disruption to schooling but recognised the potential value in still gathering post-intervention data for exploring the impact of COVID-19 in a second school year. While getting into schools at the end of the 2021 school year was difficult, given restrictions on access for outside visitors, we were able to collect the necessary data, as outlined below.

## Methodology

To investigate the impact of COVID-19 on student achievement, we compared the growth from baseline (Term 1) and 8-month follow-up (Term 4) progressive achievement test results of two cohorts (2019 and 2021) of students who were matched on demographic characteristics and baseline values. These Stage 2 (Years 3 and 4) students in 101 NSW government schools formed the control group for our RCT examining the effects of Quality Teaching Rounds professional development (QTR) on student achievement (Gore et al., 2021b; Miller et al., 2019). Using control group data ensured the intervention had no bearing on the outcomes of this study. In 2019, we collected pre- (Term 1) and post- (Term 4) intervention data from 2,063 students in 62 control group schools. In Term 1, 2021, we recommenced a second cohort within the RCT which had been postponed in 2020 when it was clear the intervention could not go ahead because of COVID-19. We collected pre-intervention baseline data from 1764 students in 39 control group schools before the emergence of the Delta variant in Australia caused another, longer period of lockdown (Table 1). Schools reopened partway through Term 4, 2021, with just enough time to collect the follow-up data before the school year ended.

**Table 1:** Learning from home, NSW government schools, 2020 and 2021

Year	Learning from home commences	Return to in-classroom learning	Period of school closure
2019	N/A	N/A	0 weeks
2020	Monday 23 March	Monday 25 May	9 weeks^1^
2021	Monday 28 June	Monday 25 October	16 weeks^2,3^

^1^Includes NSW school holidays

^2^Includes NSW school holidays. A phased return to school commenced on 18 October (Kindergarten, Year 1, and Year 12); 25 October (all other Years)

^3^Restrictions in NSW varied by region. In this study, students from 57 schools did not attend school for the full 16 weeks, 11 schools for 11 weeks, 4 schools for 10 weeks, 1 school for 8 weeks, 5 schools for 7 weeks, and 2 schools for 6 weeks

### Student achievement

Students in both the 2019 and 2021 cohorts completed Progressive Achievement Tests (PATs) in mathematics and reading (Australian Council of Educational Research [ACER], 2011) in Term 1 and Term 4 administered by, or with the support of, trained research assistants. PATs are used by more than 6000 schools and 1.5 million students annually. The validity and reliability of these tests have been well established with usage across multiple years and countries to assess and monitor students’ skills, understandings, and growth over time (Fogarty, 2007). Item reliability produced using Rasch modelling is reported at 0.87–0.91 (Lindsey et al., 2010), with this measure interpreted in the same manner as Cronbach’s alpha within classical test theory (Bond et al., 2020). Percentile scores were used to allow for comparison of students across test levels (Year 3, Year 4) and testing years (2019, 2021). For ease of reading, hereafter, we refer to Year 3 and 4 as ‘grades’ and the calendar years as ‘years’.

### Index of community socio-educational advantage

In order to investigate differential effects for students experiencing disadvantage, we used the national Index of Socio-Educational Advantage (ICSEA). As the name suggests, ICSEA is a scale of socio-educational advantage which is calculated for every Australian school (ACARA, 2020b). Created by ACARA, it enables researchers, policymakers, school leaders, teachers, parents, and students to make comparisons between schools. ICSEA takes into account student factors (parental occupation and education) and school factors (geographical location and proportion of Indigenous students at the school). Calculated on a scale, with a median of 1000 and a standard deviation of 100, ICSEA values typically range from 500 (extremely disadvantaged) to 1300 (extremely advantaged). In NSW, the state in which this study was undertaken, ICSEA values in government primary schools range from 589 to 1189 (NSW Government, 2022). ICSEA was used as a continuous variable during matching and analysis; however, for the sake of interpretation, schools were grouped into three ICSEA categories, low (< 950), mid (950–1050), and high (1050 >). These categorical cut-points represent half a standard deviation on either side of the national mean (± 50) to create categories around a group of average schools near the mean of 1000. This approach has previously been applied using national testing data in quarter standard deviation units (Goss, Emslie, & Sonnemann, 2018).

### Sample and analysis

Students from 39 schools participated in the study during 2021. The data from this cohort were compared with data collected from the 62 government schools in 2019. Schools that participated in 2019 were primarily located in major cities (*n* = 35) and regional areas (inner regional, *n* = 21; outer regional, *n* = 5). One school was in a very remote area. A similar pattern characterised schools that participated in 2021, with most in major cities (*n* = 33) and a smaller group in regional areas (inner regional, *n* = 5; outer regional, *n* = 1). There were no schools from remote or very remote communities in the 2021 sample.

To ensure the robustness of the comparison between 2019 and 2021, we used a combination of student and school variables to match participants from the two cohorts. Student variables were grade, gender, Indigenous status, and baseline achievement. School variables were class baseline achievement (class mean), school ICSEA, ICSEA category, and school location (urban or regional, with the latter including further subcategories of inner regional, outer regional and very remote). Our analysis rests on the assumption that students with the same demographic characteristics and starting points should display equivalent achievement growth under normal circumstances.

Two sets of matched data were created – a “basic” match and a “strict” match. For the basic match, we found at least one 2019 student for every 2021 student by using the following cascading series of criteria, stopping when at least one match was found:

1. Categorically matched on year level, baseline test score within one percentile, and numerically matched on ICSEA category, ICSEA within 25, location, and baseline class average score within 10 percentiles.
2. The same as 1, except removing the numerical match on class average score within 10 percentiles.
3. The same as 2, except removing the categorical match on location.
4. The same as 3, except removing the categorical match on ICSEA category.
5. The same as 4, except ICSEA within 25 is eased to find the closest ICSEA value (and select one at random if two or more students have the same ICSEA value distance).
6. The same as 5, except the baseline test score within one percentile is eased to find the student with the smallest distance metric where the distance metric is calculated as: |ICSEA_2021_ – ICSEA_2019_|/25 +|PercentileScore_2021_ – PercentileScore_2019_|/1.

The strict match applied the demographic criteria more stringently to investigate if this more sensitive analysis produced a different pattern of results. For the strict match, any student who could not be matched on the full set of criteria was discarded from the analysis. To be an eligible 2019 student, categorical matches on year level, gender, Indigenous status, ICSEA category and location were required, as well as numerical matches on ICSEA within 25, baseline score within one percentile and class average baseline score within 10 percentiles.

Linear mixed models were fitted to compare student achievement outcomes for the two cohorts (2019 and 2021). Year (2019 and 2021), time (Baseline [Term 1] and 8-month follow-up [Term 4]), and year-by-time interactions were assessed as categorical fixed effects within the models. School ICSEA was included as a covariate. To account for the two time points for each individual, a repeated measures statement was included (unstructured covariance matrix). Random intercepts were included to account for the hierarchical nature of the data (students within classes within schools). Differences in means and 95% confidence intervals (CIs) were determined using these models. The 2019 cohort was set as the reference group for all comparisons and significance was set at *p* < 0.05.

Investigation of achievement differences between cohorts across the spectrum of socio-educational advantage was addressed in three ways: 1) a three-way interaction (year-by-time-by-ICSEA) was included as a fixed effect to assess if the linear interaction between ICSEA and achievement growth varied by cohort; 2) a three-way interaction (year-by-time-by-ICSEA category) was included as a fixed effect to assess if achievement growth between cohorts was comparable across ICSEA categories; and 3) subgroup analysis was conducted to investigate if achievement growth differed by cohort within each ICSEA category (low < 950, mid 950–1049, and high 1050 +).

As the categorical interaction and subgroup analyses involved multiple comparisons, *p*-values were corrected using Sidak adjustment to avoid issues of multiplicity. Given the < 950 group was most affected during the initial year of disrupted schooling (Gore, Fray et al., 2021), it was chosen as the reference group for comparisons. Cohen’s (1988) *d* was used to determine effect sizes (*d* = (Mchange2021 – Mchange2019)/σ pooled, where Mchange is the change in mean score for each group relative to its baseline value and σ is the pooled unconditional standard deviation).

### Cohort demographics

Where possible, all 2021 students were matched to 2019 students. Table 2 displays the stage of the matching procedure at which students were matched. The majority of students, 61% for mathematics and 64% for reading, were matched without the release of any matching criteria. Only 2% of students (*n* = 35) required the release of the baseline result match (within one percentile) for a suitable match to be found for mathematics, and 3% of students (*n* = 37) for reading.

**Table 2:** Basic matching hierarchy—Mathematics and Reading

Match type	Mathematics (%)	Reading (%)
Basic match	61	64
Dropped class average within ten points	14	12
Dropped categorical location match	10	9
Dropped categorical ICSEA match	2	2
Ease ICSEA to nearest match	10	12
Ease percentile to nearest result	2	3
Total	100	100

Basic matching produced a well-balanced sample (Tables 3 and 4), with demographics closely aligned between the two cohorts. Baseline results for mathematics and reading were nearly identical for the 2019 and 2021 cohorts. Strict matching captured ~ 50% of the 2021 participants (Tables 3 and 4). As anticipated due to the stringent matching on criteria, this more sensitive analysis produced near identical demographic values for gender, Indigenous status, location, and ICSEA, as well as for baseline mathematics and reading values between the cohorts. There were marginal differences in location across cohorts due to the combination of regional and rural categories to produce the regional category for matching. Inspection of the demographic characteristics between the basic and strict matches shows a reduction in the proportion of Indigenous and regional students in the sample, and slightly higher ICSEA. The small proportion of Indigenous students and regional schools and the requirement to match with a student within one percentile from a class within 10 percentile points limited matching among these students.

**Table 3:** Student sample characteristics by Year—Mathematics

Data	Valid data		Basic match		Strict match	
2021 students matched	N/A		100%		48%	
	2019	2021	2019	2021	2019	2021
Sample (*n*)	2,376	1,422	1,422	1,422	686	686
Female (%)	49	50	49	50	48	48
Indigenous (%)	6	7	5	7	2	2
Regional (%)	36	16	26	16	16	16
Inner Regional (%)	27	14	19	14	10	12
Outer Regional (%)	7	2	5	2	3	4
Very Remote (%)	2	0	2	0	3	0
ICSEA (mean)	1004	1019	1020	1019	1030	1029
Baseline (mean)	41.90	46.90	46.91	46.90	47.61	47.62

**Table 4:** Student sample characteristics by Year – Reading

Data	Valid data		Basic match		Strict match	
2021 students matched	N/A		100%		51%	
	2019	2021	2019	2021	2019	2021
Sample (*n*)	2405	1420	1420	1420	725	725
Female (%)	50	50	50	50	49	49
Indigenous (%)	6	7	5	7	2	2
Regional (%)	36	16	26	16	14	14
Inner regional (%)	27	14	19	14	10	13
Outer regional (%)	7	2	6	2	2	1
Very remote (%)	2	0	2	0	2	0
ICSEA (mean)	1004	1019	1021	1019	1029	1028
Baseline (mean)	34.10	41.37	41.34	41.37	38.58	38.57

With regard to the representativeness of the sample of matched students, data from the 2020 ACARA Australian Schools List (ACARA, 2022) report the demographics of NSW primary schools (the population from which this sample is drawn) as 48% female, 7.9% Indigenous, and 46.9% regional, with a mean ICSEA of 986. The demographics of our sample are more representative of urban primary schools in the NSW government sector which have a mean ICSEA of 1026 and enrol 76% of the state’s primary students.

## Results

### Student achievement in mathematics

**Fig. 1:**
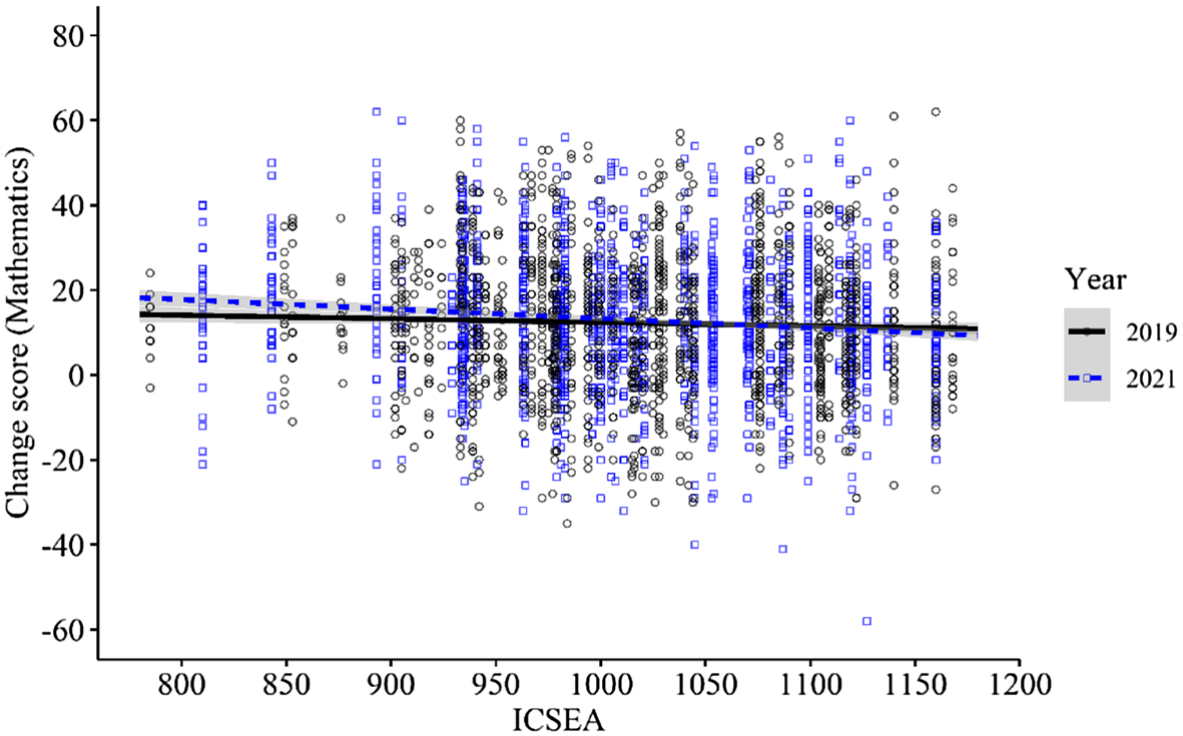
Mathematics achievement growth by cohort—Interaction with ICSEA

Mathematics achievement was equivalent between the 2021 and 2019 cohorts overall (Table 5), with less than half of one percentile point difference in the achievement gain across the 8-month retest period (difference 2021 vs 2019 = 0.48; 95% CI: − 0.73, 1.68; *d* = 0.02, *p* = 0.438). ICSEA was a significant covariate in the overall model (*p* < 0.001), and the Year x Time x ICSEA interaction demonstrated statistical significance (− 0.015; 95% CI: − 0.031, − 0.001; *p* = 0.041). Figure 1 illustrates the difference in the linear interaction between student change score in mathematics and ICSEA for the two cohorts (shaded area indicates the standard error of the plotted line). Note the negative relationship between student change scores and ICSEA is more defined (steeper slope) for the 2021 cohort. The pattern of results was more pronounced for the strictly matched data, with a significant Year x Time x ICSEA interaction (-0.028; 95% CI: − 0.052, − 0.004; *p* = 0.020).

**Table 5:** Achievement in mathematics (2019, 2021)

Match	ICSEA category	Year	Count	Baseline	8-months	Adjusted mean difference: 2021–2019 (95% CI)	Effect size	*p*
Basic	Total	2019	1,422	46.90 (27.39)	59.17 (25.25)			
		2021	1,422	46.91 (27.42)	59.41 (25.25)	0.48 (-0.73, 1.68)	0.02	0.438
	1050 +	2019	485	59.36 (25.05)	71.70 (20.92)			
		2021	562	58.22 (25.58)	69.50 (22.89)	-1.05 (-3.05, 0.93)	− 0.04	0.654
	950–1050	2019	569	43.29 (26.76)	54.95 (25.10)			
		2021	569	41.69 (26.22)	54.04 (24.65)	0.68 (-1.20, 2.57)	0.03	0.850
	< 950	2019	291	34.13 (24.35)	47.64 (23.62)			
		2021	291	35.23 (25.05)	51.60 (23.77)	2.86 (0.30, 5.41)	0.11	0.104
Strict	Total	2019	686	47.61 (26.75)	60.16 (24.17)			
		2021	686	47.62 (26.73)	60.07 (24.06)	-0.10 (-1.91, 1.71)	0.00	0.310
	1050 +	2019	422	60.92 (25.12)	72.80 (20.39)			
		2021	422	60.93 (25.12)	70.66 (21.79)	-2.15 (-4.87, 0.58)	− 0.09	0.342
	950–1050	2019	343	40.63 (24.12)	53.42 (22.57)			
		2021	343	40.64 (24.11)	53.17 (22.95)	-0.26 (-3.09, 2.58)	− 0.01	0.997
	< 950	2019	133	31.59 (21.13)	45.23 (21.59)			
		2021	133	31.61 (21.10)	50.77 (22.16)	5.52 (1.10, 9.94)	0.24	0.040

**Fig. 2:**
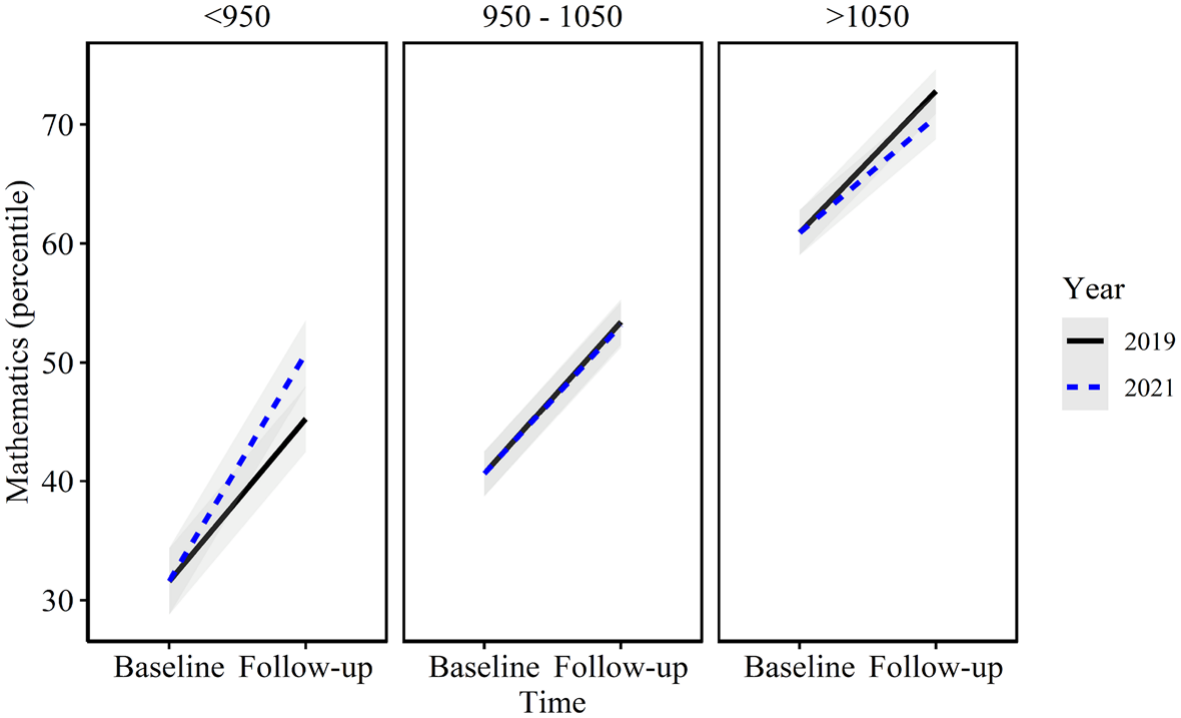
Mathematics achievement growth by cohort by ICSEA group (mean, 95% confidence interval)

When comparing the growth between cohorts and ICSEA categories (Year x Time x ICSEA category), the difference in growth between the low (ICSEA < 950) and high (ICSEA 1050 +) groups was significant (difference = 3.91 95% CI: 0.578, 7.24; *p* = 0.042), a function of the greater growth in 2021 for the low-ICSEA category and lower growth in 2021 for the high-ICSEA category. Figure 2 displays the interaction across the ICSEA categories. There was no significant difference between low- and mid-ICSEA categories.

When examining student achievement within ICSEA categories (subgroup analysis), a more nuanced picture emerged which elaborates the demonstrated interaction of achievement growth with ICSEA (Table 5). As illustrated in Fig. 2, in 2021, students from low-ICSEA schools (ICSEA < 950) achieved greater but non-significant growth in mathematics than students in the same ICSEA category in 2019 (difference = 2.86; 95% CI: 0.30, 5.41; *d* = 0.11; *p* = 0.104). Students from mid-ICSEA schools displayed no difference in achievement growth between cohorts, while students in the high-ICSEA category achieved less growth in 2021 than in 2019. This trend, using the strictly matched data (Table 5), shows a slightly stronger, and significant, effect in the < 950 ICSEA group for mathematics achievement.

### Student achievement in reading

**Fig. 3:**
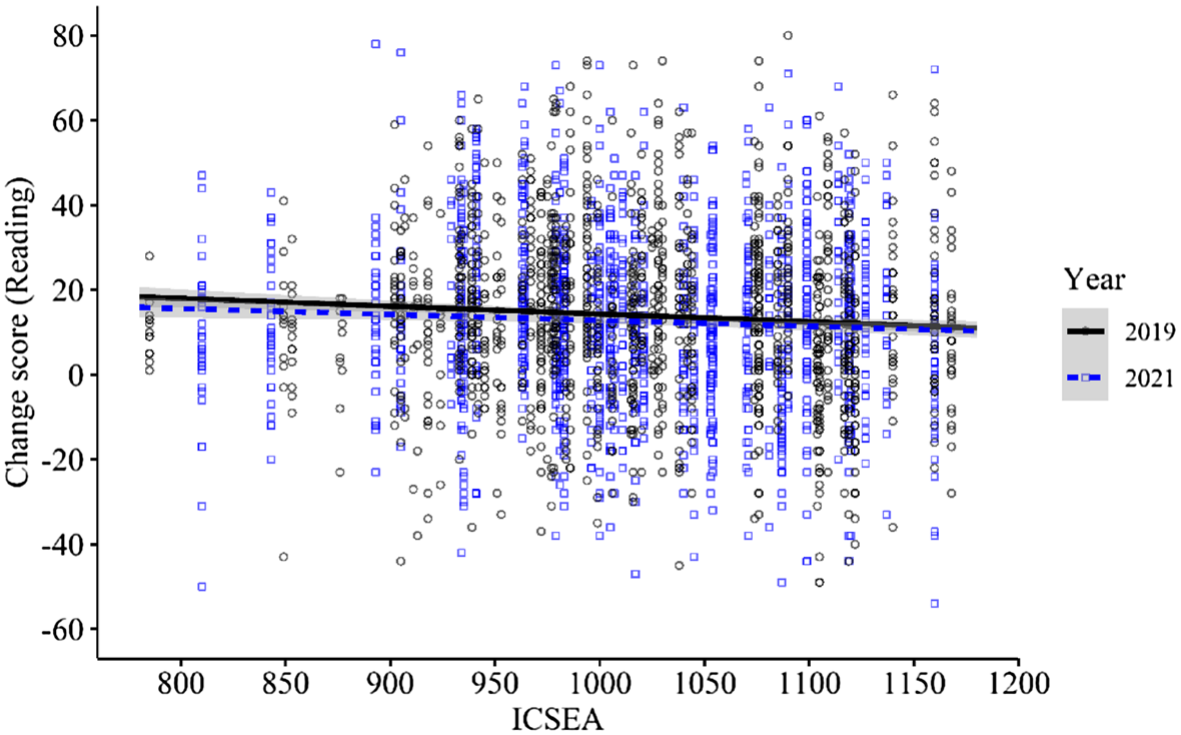
Reading comprehension achievement growth (2019, 2021)—Interaction with ICSEA

Overall, there was a marginal, but insignificant, difference in the reading achievement growth of the two cohorts (difference 2021 vs 2019 = − 1.23 95% CI: − 2.74, 0.27;* d* = − 0.04;* p* = 0.107) (Table 6). While ICSEA was a significant covariate in the overall reading model (*p* < 0.001), the Year × Time × ICSEA interaction was not statistically significant (0.002; 95% CI: − 0.017, 0.021; *p* = 0.832). Figure 3 displays the interaction between student change scores and ICSEA for each cohort. As opposed to the mathematics interaction, the 2021 cohort displays an almost identical relationship between reading growth and ICSEA as the 2019 cohort.

**Table 6:** Achievement in reading (2019, 2021)

Match	ICSEA category	Year	Count	Baseline	Gain	Adjusted mean difference: 2021–2019 (95% CI)	Effect size	*p*
Basic	Total	2019	1,420	41.35 (29.17)	55.24 (26.70)			
		2021	1,420	41.37 (29.18)	54.03 (27.37)	-1.23 (-2.74, 0.27)	− 0.04	0.107
	1050 +	2019	503	54.12 (28.99)	65.25 (24.98)			
		2021	561	52.16 (28.63)	63.26 (25.15)	-0.33 (-2.53, 2.46)	0.00	0.999
	950–1050	2019	632	36.61 (27.56)	52.46 (26.25)			
		2021	567	36.11 (27.90)	49.35 (27.35)	-2.61 (-4.92, -0.30)	− 0.09	0.078
	< 950	2019	285	29.29 (24.38)	43.71 (24.52)			
		2021	292	30.88 (25.91)	45.39 (26.45)	0.10 (-3.09, 3.30)	0.00	0.999
Strict	Total	2019	725	38.58 (29.07)	53.46 (27.23)			
		2021	725	38.58 (29.09)	52.09 (26.94)	-1.36 (-3.47, 0.74)	− 0.05	0.205
	1050 +	2019	289	53.50 (28.41)	64.69 (24.26)			
		2021	289	53.53 (28.38)	63.53 (24.71)	-1.18 (-4.75, 2.38)	− 0.04	0.861
	950–1050	2019	318	29.97 (25.57)	47.72 (26.83)			
		2021	318	29.94 (25.60)	45.10 (26.15)	-2.59 (-5.62, 0.44)	− 0.08	0.289
	< 950	2019	118	25.23 (23.26)	41.41 (25.30)			
		2021	118	25.22 (23.26)	42.92 (24.34)	1.52 (-3.11, 6.15)	0.06	0.918

**Fig. 4:**
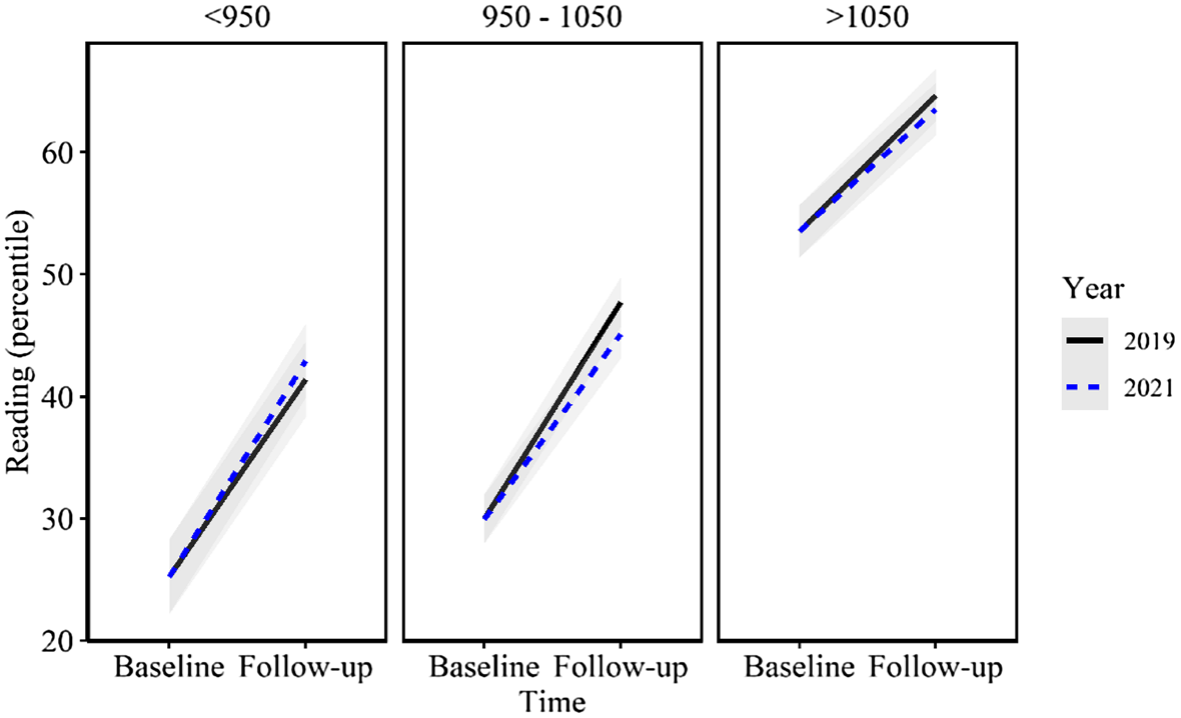
Reading comprehension achievement growth by cohort by ICSEA group (mean, 95% confidence interval)

When comparing the growth between cohorts and ICSEA categories (Year x Time x ICSEA category), there were no significant differences in growth between any of the ICSEA categories (Fig. 4). Likewise, when investigating achievement of the cohorts within ICSEA bands, there were no significant differences between 2019 and 2021 students in any of the ICSEA categories, and there was no linear pattern to the results. The only discernible pattern to the results among ICSEA categories was that the mid (ICSEA 950–1050) and high (ICSEA > 1050) categories displayed negative results for the 2021 cohort in relation to 2019, while the low (ICSEA < 950) group displayed slightly positive results for the 2021 cohort.

## Discussion

Despite many predictions of major short- and long-term ‘learning loss’ as a result of school closures (Engzell et al., 2021; Lewis et al., 2021) and robust empirical evidence of learning loss in some countries (Donnelly & Patrinos, 2021; Hammerstein et al., 2021), our study found no average decline in student achievement in mathematics or reading when compared to student achievement in 2019. After eight weeks of learning from home in 2020, 14 weeks in 2021, and all the associated challenges to schooling and society brought about by the pandemic, negative effects on student learning in NSW primary schools were minimal in our sample.

### Unexpected results

Surprisingly, we found significant positive effects on mathematics achievement for students in disadvantaged schools. This result stands in stark contrast with predictions that COVID-19 would lead to significant loss in learning that would disproportionately impact the most vulnerable students (Brown et al., 2020; Schleicher, 2020; Sonnemann & Goss, 2020). While, on the surface, our result is counterintuitive, other studies conducted during the first affected school year also reported minimal impact on student learning (Meeter, 2021; Schult et al., 2022; Tomasik et al., 2021).

Our own study of the impact of COVID-19 on student learning in 2020, using similar methodology, found minimal impact overall, but two months’ less achievement growth in mathematics in 2020 than in 2019 for Year 3 students from low-ICSEA schools (Gore, Fray et al., 2021). A year later, we still found minimal impact overall, but the positive result for disadvantaged students—approximately three months’ additional growth in mathematics using conventions for reporting effect sizes adopted by the Education Endowment Foundation (2018)—is striking. How might the results overall and the turnaround in mathematics achievement for students in the least advantaged schools be explained? Several factors are worth consideration.

First, schools, teachers, and students were clearly more prepared for learning from home in Term 3, 2021 than in Term 2, 2020. Teachers, students, and their families gained experience with online technologies and how to engage in online learning—activities that were completely unfamiliar to most when the pandemic struck in 2020 (Fray, Jaremus, Gore, Miller, et al., 2022). New ways of teaching were developed to ensure student learning continued. In addition, acting on deep concern for the most vulnerable in our communities, school leaders and teachers provided targeted support to the most disadvantaged students, made possible by a raft of initiatives designed to support student learning in literacy and numeracy, supported by substantial new funding ($720 million over two years for the tutoring programme alone) (NSW Government, 2021d).

Second, timing may have also played a role. The tests we used are designed to assess achievement growth across the entire school year. All students completed baseline testing in Term 1, and follow-up data collection in Term 4. In 2020, schools closed in Term 2 while, in 2021 they closed in Term 3. It is possible that the in-school experience during the first two terms of 2021 helped students catch up on any learning they may have missed during 2020 and even get ahead. In the context of widely held concerns about learning loss for disadvantaged students (Sonnemann & Goss, 2020), it is possible that intensive efforts to bolster strong learning outcomes paid off.

Third, students in the lower ICSEA schools may have spent more time in school than those in mid- and high-ICSEA schools. In place of the state- or nation-wide lockdowns that characterised 2020, lockdowns in 2021 were based on the number of COVID-19 cases reported in local government areas. Our tracking of closures for the schools involved in our study shows that several schools, particularly those in regional areas, were able to remain open for longer. Students in low-ICSEA schools spent an average of 8.8 weeks learning from home, which was three weeks less than in mid-ICSEA schools (11.75 weeks) and five weeks less than in high-ICSEA schools (14 weeks). The additional time in face-to-face schooling may, in part, account for the positive results observed for students in low-ICSEA schools in relation to their counterparts in mid- and high-ICSEA groups.

Fourth, arguably, the intense focus on literacy and numeracy outcomes as the building blocks of broader academic success may have narrowed the curriculum as teachers grappled to catch up on presumed missed learning (Fray, Jaremus, Gore, Miller et al., 2022). Such efforts were fervent, especially prior to the release of the 2021 NAPLAN results which provided some comfort that learning in mathematics and literacy had not been compromised to any great extent.

On a cautionary note, however, the privileging of mathematics and literacy learning may mask unintended negative effects of COVID-19 on student learning in other subjects, such as physical education, the arts, and music (Broerse, 2021; Brown et al., 2020; Cruickshank et al., 2021). The goals for education in Australia emphasise the development of the whole child (Department of Education Skills & Employment, 2019). We ask: At what cost does a narrow focus on success in maths and reading come during post-pandemic recovery, and how does such success impact the longer-term development of the children involved? More broadly, perhaps we need to temper celebrations of growth in academic achievement if it has come at the expense of negative effects on student wellbeing (Batchelor et al., 2021; Fray, Jaremus, Gore & Harris, 2022; Institute for Social Science Research [ISSR], 2020).

### COVID-19 as a catalyst for greater equity

In Australia, little has changed in the 10 years since the release of the Gonski review of school funding (Gonski et al., 2012) which was heralded as a blueprint for delivering more equitable schooling and improving student performance. The failure of successive governments to implement the report’s recommendations and adequately fund schools continues to impact the most disadvantaged students. While the growth in achievement by students in low-ICSEA schools (ICSEA < 950) is to be celebrated, their attainment remains significantly lower than that of students in high-ICSEA schools, as depicted in Fig. 2. Australia languishes in the bottom-third of the world’s wealthiest countries (30th of 38) when it comes to equality in education (UNICEF Office of Research, 2018). The results reported here might demonstrate what *can* be done in the quest for greater equity in schooling when the policy commitment to equity is matched by practical action and funding initiatives that deliver better outcomes.

### Limitations

Some limitations of our study should be considered when interpreting the results. First, the sample size (*n* = 3827 Year 3 and Year 4 students from 101 schools) is relatively small, given the primary school population in NSW of 499,725 students in 1775 schools (NSW Government, 2022). The rigorous matching of 2021 and 2019 students across a diverse range of schools increases the validity of findings. Second, while our analysis shows that students from low-ICSEA schools achieved an additional three months’ growth in mathematics, we acknowledge that not all students in low-ICSEA schools are from disadvantaged backgrounds. MySchool^1^ data for our sample indicate that a large proportion (between 82% and 95%) of students from each school in the low-ICSEA band were from the bottom two quartiles for socio-educational advantage. Third, the data were originally gathered for a different study rather than collected specifically to investigate the effects of the pandemic. Nonetheless, they provide rigorous comparable evidence of changes in student achievement following two consecutive years of disrupted schooling. Finally, while the standardised Progressive Achievement Tests in mathematics and reading comprehension we used as our measures of student achievement have good predictive validity (Fogarty, 2007), they do not assess student achievement across the entire curriculum—a common limitation of standardised testing. Without these data, however, we would have no robust evidence with which to explore the effects of the pandemic. We look forward to similar analyses conducted in other nations, which will be critical to understanding how contextual factors and different experiences of the pandemic have affected student achievement over time.

## Conclusion

This study offers some of the earliest evidence, globally, on how the COVID-19 pandemic affected student achievement after two consecutive years of disrupted schooling. Given the somewhat surprising finding that students from disadvantaged schools are now doing better in mathematics and no worse in reading than their counterparts in 2019, we contend the pandemic may have been a catalyst for practical actions of a kind that could rectify longstanding inequalities in schooling in Australia. We deliberately use tentative language here because, to date, there is no rigorous systematic evidence showing the effects on student achievement of the various government initiatives implemented during the pandemic.

Perhaps it took a pandemic and dire warning about the future of the nation, to finally motivate investment of a kind that can begin to narrow the enduring achievement gap between advantaged and disadvantaged students. Based on the evidence from our study, we argue that it is possible to enhance outcomes for students in disadvantaged schools, and to arrest cycles of disadvantage, if the rhetoric of increasing equity is matched by effective strategies (OECD, 2018; Sahlberg, 2019; Zyngier, 2012). If governments continue to support the learning of students who are most in need, we might begin to lift our relative national performance in both excellence and equity on the international stage. Most importantly, we might make a real difference to the lives of students, families, and communities who continue to suffer the negative effects of inequitably funded schooling on top of broader structural injustices.

## Data Availability

Not applicable.

## Footnotes

1 MySchool is a website resource administered by ACARA which compiles profile and demographic characteristics of every school in Australia.
