# Correction to: Was COVID-19 an unexpected catalyst for more equitable learning outcomes? A comparative analysis after two years of disrupted schooling in Australian primary schools

**DOI:** 10.1007/s13384-023-00624-w

**Published:** 2023-03-21

**Authors:** Andrew Miller, Leanne Fray, Jennifer Gore

**Affiliations:** Teachers and Teaching Research Centre, School of Education, University of Newcastle, Callaghan, Australia

**Correction to: The Australian Educational Researcher** 10.1007/s13384-023-00614-y

In the original publication of the article, the abstract was incorrectly published.

The correct abstract is given below.

**Abstract**

By the end of 2021, more than 168 million students across the globe had missed a year of face-to-face schooling due to the COVID-19 pandemic. In NSW, Australia, most students engaged in learning from home for 8 weeks during 2020 and a further 14 weeks during 2021. This study provides robust empirical evidence on how two years of disruptions to schooling affected student learning. Drawing on matched data for 3827 year 3 and 4 students from 101 NSW government schools, this paper compares student achievement growth in mathematics and reading for 2019 (pre-pandemic) and 2021 (second year of the pandemic) student cohorts. While overall there was no significant difference between cohorts, when analysed by socio-educational advantage, we were surprised to find that students in the lowest band achieved approximately 3 months’ additional growth in mathematics. Arguably, grave concerns about the potentially dire impact of COVID-19 on the learning of disadvantaged students were met by investments that made a difference. We argue that targeted funding and system-wide initiatives to support more equitable outcomes should remain a priority after the pandemic if Australia is to meet its aspirations for excellence and equity.

The original article has been corrected.

